# Review of "*Acanthamoeba: Biology and Pathogenesis*" by Naveed Ahmed Khan

**DOI:** 10.1186/1756-3305-2-16

**Published:** 2009-03-28

**Authors:** Fiona L Henriquez

**Affiliations:** 1 School of Engineering and Science, University of the West of Scotland, 1 High Street, Paisley, PA1 2BE, UK

## Review

There is an increasing interest and awareness of the free-living amoeba, *Acanthamoeba*, over recent years as an opportunistic pathogen of medical importance. The publication of this book is a timely reflection of this current situation. The author is to be congratulated on the provision of such a comprehensive review of the literature concerning all aspects of *Acanthamoeba *research. The intention of the author was to provide an essential reference for researchers of infectious diseases. This is achieved and in addition, this book should also be beneficial to students at the university level with a specific interest in microbiology.

The book is divided into nine sections: Biology and Phylogeny, Life Cycle and Genotyping, *Acanthamoeba *Infections, Pathogenesis, Immune Response, Strategies Against *Acanthamoeba *Infections, *Acanthamoeba*: Trojan Horse of the Microbial World, Conclusion and Future Studies, Bibliography. Each section is appropriately detailed, complete and subdivided into specific topics. One minor criticism would be that the diagrams and illustrations are not always clear, perhaps due to many having been reproduced from other sources.

The first two sections detail the cell biology and the habitat of the *Acanthamoeba *from its organelles to their functions, from its distribution to the relationship it has developed with the environment. This section is very informative on its life cycle and allows the reader to understand how this organism is now known to be so versatile and adaptable. Interestingly, it also provides useful information in culturing *Acanthamoeba *under laboratory conditions, such as isolating *Acanthamoeba *from environmental samples and genotyping the species, successful axenic cultivation, methods of encystation, storage and determination of the viability of trophozoites and cysts. These are essential tools for researchers working with this organism.

This middle sections concern the increasing knowledge we have acquired of the infections that *Acanthamoeba *can cause as an opportunistic pathogen. The increase in incidence of *Acanthamoeba *keratitis with the use of contact lenses has been a major contributing factor in the growing awareness of this pathogen. The author details with clarity how *Acanthamoeba *can bind to both worn and unworn contact lenses, the impact of biofilm in contracting *Acanthamoeba *keratitis, the clinical outcome of infection and diagnosis. A significant chapter is dedicated to *Acanthamoeba *granulomatous encephalitis (GAE) which, although a rare disease, is often fatal. Major concerns regard immunocompromised patients, who are rendered more susceptible to this disease. A smaller section focuses on cutaneous acanthamoebiasis, which is characterised by nodules in the skin and ulcerations. For both GAE and cutaneous acanthamoebiasis there is no recommended treatment. This book raises awareness of these diseases that often are misdiagnosed.

Attention is also paid to the different models used in research to understand pathogenesis, from the *in vivo *models for *Acanthamoeba *keratitis, which include the Yucatan micropig and Chinese hamster to those for GAE, which include the use of the African migratory locust. However, many studies that dissect the host-pathogen relationship have often used *ex vivo *and *in vitro *techniques to understand pathogenesis at the molecular level. Host-pathogen interaction has been elaborately described in this book from the crossing of the biological barriers to direct virulence factors, including contact-dependent and -independent mechanisms and indirect virulence factors, such as temperature tolerance, osmotolerance and pH tolerance. This suitably introduces the section, which focuses on the host's immune response to infection of this organism. The introduction to the immune system provided in this text is simplistic, but it allows the reader to acquire to the basic concepts necessary to understand the immune response against *Acanthamoeba *infections and what mechanisms *Acanthamoeba *uses to evade immune responses.

The final sections focus on the strategies currently in place against *Acanthamoeba *infections and the author provides an excellent summary of a plethora of chemotherapeutic agents that have been tested for anti-Acanthamoebic activity. These include membrane-acting agents, inhibitors of DNA synthesis and polyamine metabolism, RNA synthesis inhibitors, protein synthesis inhibitors, tricyclic neuroleptic agents and antimicrobial compounds from natural products. Despite the large variety of anti-Acanthamoebic compounds, emerging drug resistance is a problem and focus is moving towards prevention of infection with the use of disinfectants. This problem is dutifully highlighted in this book where the author also describes future prospects for treatment and prevention.

*Acanthamoeba *is often referred to as the 'Trojan Horse' of the microbial world. The significance of this is illustrated very well in this book, as the author explains how *Acanthamoeba *can habour a diverse number of pathogens, including viruses, bacteria, yeasts and Protozoa, thus making it not only an opportunistic pathogen, but also a vector for other important pathogens.

This book is certainly a 'must read' for all scientists interested in medical and environmental microbiology. It is a very convincing overview and foundation of what is already known about *Acanthamoeba*, but the literature is constantly progressing rapidly and new information is arising about this, until recently, understudied organism.

## Competing interests

The author declares that they have no competing interests.

